# Surface area wiped, product type, and target strain impact bactericidal efficacy of ready-to-use disinfectant Towelettes

**DOI:** 10.1186/s13756-018-0416-z

**Published:** 2018-10-11

**Authors:** Alyssa M West, Carine A Nkemngong, Maxwell G Voorn, Tongyu Wu, Xiaobao Li, Peter J Teska, Haley F Oliver

**Affiliations:** 1 Department of Food Science, Purdue University, West Lafayette, IN 47907 USA; 2 Diversey Inc., Charlotte, NC 28273 USA; 3 Department of Food Science, Purdue University, 745 Agriculture Mall Drive, West Lafayette, IN 47907 USA

**Keywords:** Disinfectant, Towelette, Efficacy

## Abstract

**Background:**

Disinfectant products are often used on environmental surfaces (e.g. countertops, patient beds) and patient care equipment in healthcare facilities to help prevent the transmission of healthcare-associated infections. Ready-to-use (RTU) disinfectants in the form of pre-wetted towelettes are increasingly popular among healthcare facilities. Currently, the EPA does not require disinfectant manufacturers to include a recommended maximum surface area per towelette on their product labels. The objective of this study was to investigate the efficacy of disinfectant towelette products on a hard non-porous surface across different coverage areas using a quantitative EPA method. We hypothesized that there would be significant differences in the efficacy of disinfectant towelette products, and that the greater surface area(s) wiped would result in reduced bactericidal efficacy.

**Methods:**

This study tested ten disinfectant towelette products against *Staphylococcus aureus* strain ATCC CRM-6538 and *Pseudomonas aeruginosa* strain ATCC 15442 on Formica surfaces. Defined surface areas were wiped and the towelette weighed before and after wiping to determine the amount of liquid released. Bactericidal efficacy testing was also performed after wiping following standard EPA protocols.

**Results:**

We found that disinfectant product, area of surface wiped, and strain impacted the bactericidal efficacy achieved. Disinfectant product type and area of surface wiped significantly impacted the percent of liquid released per ft^2^from the towelette.

**Conclusion:**

Overall, bactericidal efficacy varied by towelette product, surface area wiped, and strain. This study also found that wiping larger surface areas may lead to decreased bactericidal efficacy. Further research is needed to test its implication.

## Background

Healthcare-associated infections (HAIs) are reported to occur in one out of 25 patients every day in the United States [1]. It is estimated that in 2011, 721,000 cases of HAIs occurred in United States acute care hospitals [2]. Healthcare-associated pathogens can colonize a vast array of environmental surfaces and patient care equipment in healthcare facilities and transmission from these surfaces to patients can lead to HAIs [3, 4]. Disinfectant products are often used on environmental surfaces (e.g. countertops, patient beds) and patient care equipment in healthcare facilities to help prevent the transmission of healthcare-associated pathogens. Although disinfectants are generally accepted to be effective against a wide range of pathogens, major factors exist that can cause differences and reductions in bactericidal efficacy. For example, disinfectant concentration can affect the bactericidal efficacy achieved. Concentrations that are lower than the label-use have been shown not to be as effective [5–9]. Reduced contact times (as compared to label instructions) can lower a product’s efficacy as well [5–9]. Furthermore, a product’s overall formulation and active ingredients play a role in bactericidal efficacy. Previous work by our group demonstrated that a sodium hypochlorite-based disinfectant product was significantly more effective against multiple MRSA strains than a quaternary ammonium compounds (quat)- based product [5]. Other published studies have also shown that disinfectants with differing active ingredients have different bactericidal efficacies against the same microorganism [6, 7, 10, 11]. Thus, understanding factors that can reduce bactericidal efficacy are important to help understand how to optimize the performance of a disinfectant.

Ready-to-use (RTU) disinfectants in the form of pre-wetted towelettes are increasingly popular among healthcare facilities. A 2014 study determined that the use of RTU disinfectant towelette products led to a faster disinfection process, higher compliance with disinfection standards, and overall cost savings as compared to traditional disinfection methods [12]. The CDC specifically recommends using an Environmental Protection Agency (EPA)-registered disinfectant product for environmental cleaning in healthcare facilities [6]. The current EPA methodology used to register a disinfectant towelette product is the qualitative AOAC Germicidal Spray Products as Disinfectants Test modified for towelettes [13]. This protocol only requires testing of small carriers (25 mm × 75 mm glass slides) as opposed to larger surfaces areas that are more representative of actual product usage. Currently, the EPA does not require disinfectant manufacturers to include a recommended maximum surface area per towelette on their product labels. Thus, there are a number of important application considerations that are not informed by the current EPA wipe testing method.

To our knowledge, there are no prior peer-reviewed studies that have examined the effectiveness of towelettes over defined coverage areas typical of healthcare facilities. The objective of this study was to investigate the efficacy of disinfectant towelette products on a hard non-porous surface across different coverage areas using a quantitative EPA method. We hypothesized that (i) there would be significant differences in the efficacy of disinfectant towelette products against both *S. aureus* and *P. aeruginosa* and (ii) the greater surface area(s) wiped, the less bactericidal efficacy.

## Methods

### Disinfectants, bacterium, and surface used in study

This study tested ten disinfectant towelette products described below (Table 1). Diversey EasyWipes wetted with phosphate buffered saline (PBS; 4.85 mL per EasyWipe towelette) were used as a control. *S. aureus* strain ATCC CRM-6538 and *P. aeruginosa* strain ATCC 15442 were used to measure towelette disinfectant efficacy. These strains were chosen as both are currently required by the EPA to be used in testing base disinfectant efficacy claims for EPA registration [14]. Additionally, *S. aureus* is the second most common pathogen associated with HAIs [2]. The surfaces used for testing were sheets of Formica cut down to size as detailed below.

**Table 1:** Active ingredients and contact times for disinfectant towelettes tested in this study

Disinfectant Product “Name” (used throughout manuscript) ^a^	Disinfectant Active Ingredient(s)	Label Contact Time^b^
0.5% quat + 55% alcohol	- 0.25% n-alkyl dimethyl ethylbenzyl ammonium chlorides - 0.25% n-alkyl dimethyl benzyl ammonium chlorides - 55% isopropyl alcohol	2 min
0.76% quat + 22.5% alcohol	- 0.76% didecyldimethylammonium chlorides - 7.5% ethanol - 15% isopropyl alcohol	1 min
1.4% hydrogen peroxide	- 1.4% hydrogen peroxide	1 min
1.312% sodium hypochlorite	- 1.312% sodium hypochlorite	1 min
0.5% hydrogen peroxide	- 0.5% hydrogen peroxide	1 min
0.55% sodium hypochlorite	- 0.55% sodium hypochlorite	30 s
0.28% quat	- 0.14% n-alkyl dimethyl ethylbenzyl ammonium chlorides - 0.14% n-akyl dimethyl benzyl ammonium chlorides	3 min
0.21% quat	- 0.105% n-alkyl dimethyl ethylbenzyl ammonium chlorides - 0.105% n-alkyl dimethyl benzyl ammonium chlorides	3 min
0.61% quat + 56% alcohol	- 0.61% dodecyl dimethyl ammonium chloride - 27.3% ethyl alcohol - 28.7% isopropyl alcohol	1 min
0.308% quat + 21% alcohol	- 0.154% n-alkyl dimethyl benzyl ammonium chlorides - 0.154% n-alkyl ethylbenzyl ammonium chlorides - 21.000% isopropyl alcohol	2 min

^a^Abbreviated naming scheme reflects aggregated active ingredients for commercially available EPA registered disinfectants used in this study;

^b^Defined label contact time for *S. aureus* and *P. aeruginosa*

### Towelette disinfectant load and surface coverage measurements

The initial amount of liquid loaded on the disinfectant wipes was determined based on a modified technique that was used in an EPA efficacy study of sporicidal wipes [15]. The first towelette from each disinfectant container was discarded and the subsequent towelettes were used to ensure towelettes were fully wet. For each of the products, ten wipes were pre-weighed individually using a Mettler-Toledo AG204 analytical balance (accurate to 0.01 g; Mettler-Toledo LLC, Columbus, OH). After being weighed, the towelettes were rinsed under running tap water for 30 s to remove the liquid disinfectant from the towelette. Once rinsed, the towelettes were placed in a drying oven at 37 C° for 24 h. Each wipe was individually re-weighed to determine the liquid weight loaded on each wipe.

To determine the amount of liquid disinfected deposited on a defined surface area, approximately six by seven inch towelettes were wiped across textured Formica sheet surfaces ranging from one to eight feet. Formica sheets were cut to eight ft. (approx. 243.8 cm^2^) in length and marked into one foot square areas (0.5 ft. by two ft.; approx. 929.0 cm^2^). To measure the amount of liquid disinfectant deposited on the surface, the first towelette from the disinfectant container was discarded and subsequent towelettes were used to ensure the towelettes were fully wet. Each towelette was pre-weighed on an analytical balance (Mettler-Toledo LLC, Columbus, OH). Each one ft^2^ section (approx. 929.03 cm^2^) was wiped once in a down and back pattern. The same wiping pattern was used for all products tested and all sections wiped. The towelette was weighed before and after wiping a defined number of sections: one ft^2^ (~ 929.0 cm^2^), two ft^2^ (~ 1858.1 cm^2^), three ft^2^ (~ 2787.1 cm^2^), four ft^2^ (~ 3716.1 cm^2^), five ft^2^ (~ 4645.1 cm^2^), six ft^2^ (~ 5574.2 cm^2^), seven ft^2^ (~ 6503.2 cm^2^), and eight ft^2^ (~ 7432.2 cm^2^) to determine liquid weight deposited on the surface. Each of the increasing number of sections was wiped independently using a new towelette each time (e.g. a single towelette was used on an eight square foot surface). The Formica was washed with 75% ethanol and left until dry to touch between each surface area tested. Five replicates of surface coverage testing were conducted independently for the ten disinfectant products and PBS-wetted control towelette.

### Towelette bactericidal efficacy

A modified version of the EPA SOP MB-33-00 was used to conduct bactericidal efficacy testing [16]. The surface wiping method described above was used to measured bactericidal efficacy on one, two, four, and eight ft^2^ areas. After the designated Formica surface area had been wiped, the same towelette was used to wipe a 97 mm diameter Formica disc that was independently inoculated with either 50 μL of *S. aureus* culture or 50 μL of *P. aeruginosa* culture (both approximately 5.5 log CFU following EPA MB-33-00) [16]. The Formica discs were wiped, with consistent pressure, in a circular pattern (as defined in EPA MB-33-00), and the discs were left at room temperature for the disinfectant’s label contact time [16]. After the contact time was reached, the discs were swabbed using PUR-Blue Swabs (World BioProducts, Libertyville, IL) containing 10 mL sterile HiCap neutralizing buffer. The swab samplers were vortexed for 30s to release the bacteria from the sponge and the solution was vacuum-filtered onto a membrane filter (0.2 μm pore size, 47 mm grid, individual sterile pack; Pall Corporation, Port Washington, NY) following EPA MB-33-00 [15]. TSA plates (BD Biosciences, San Jose, CA) containing the plated membrane filter were incubated for 24–48 h at 37 °C, then colonies were counted. Two positive controls were conducted by directly swabbing an inoculated Formica disc with the PUR-Blue swab sampler containing 10 mL sterile HiCap neutralizing buffer. Five biological replicates were conducted for each of the disinfectant products tested and three technical replicates were performed within each biological replicate for every surface area for testing both *S. aureus* and *P. aeruginosa*.

### Statistical analyses

SAS v. 9.4 (SAS Institute, Cary, NC) was used to perform all statistical analyses. The percent liquid in grams released from the towelette for each surface area was calculated, normalized to the amount of liquid originally loaded onto the towelette (as determined by the drying method mentioned above in grams). All bactericidal efficacy data (bacterial kill) were transformed into log_10_ reduction values for analyses. All analyses had a defined significance level of α = 0.05. Data were fitted into a generalized linear mixed model with Proc Glimmix to determine the significant factors impacting the percent of liquid expended (*n* = 800). The amount of liquid released was log transformed to maximize data fitness. Least square means comparison with Tukey-Kramer adjustment was used to determine significant differences in the percent of liquid deposited onto a surface across eight areas, ten disinfectant products (with PBS wetted wipes as the control), and combinations of their interaction. To determine the factors significantly impacting bacteria reduction, a separate generalized linear mixed model was developed, with least squares means comparison and Tukey adjustment detecting significant differences in bacteria log_10_ reduction due to area, disinfectant, strain, and their interaction (*n* = 399).

## Results

### Disinfectant product, surface area wiped, and strain significantly impacted bactericidal efficacy

**Fig. 1:**
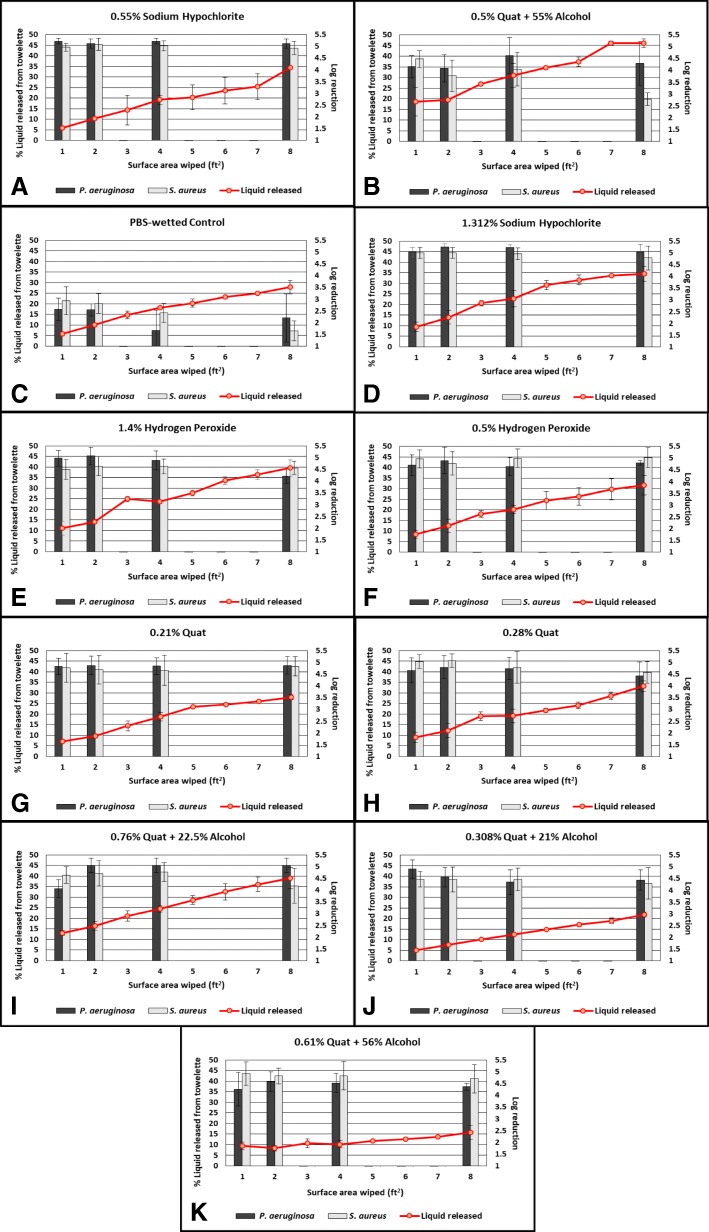
Percent of total liquid released from towelette after wiping and bactericidal efficacy against *S. aureus* and *P. aeruginosa* (expressed as log_10_ reduction values) over varying surface areas wiped for each disinfectant towelette product tested. To determine the factors significantly impacting bacteria reduction, a generalized linear mixed model was developed with least squares means comparison and Tukey adjustment detecting significant differences in bacteria log10 reduction due to area, disinfectant, strain, and their interaction (*n* = 399). Data were fitted into a generalized linear mixed model with Proc Glimmix to determine the significant factors impacting the percent of liquid expended (*n* = 800). Least square means comparison with Tukey-Kramer adjustment was used to determine significant differences in the percent of liquid deposited onto a surface across eight areas, ten disinfectant products (with PBS wetted wipes as the control), and combinations of their interaction. **a** Percent of liquid released and efficacy over varying surface areas for a 0.55% sodium hypochlorite product; **b** Percent of liquid released and efficacy over varying surface areas for a 0.5% quat product + 55% alcohol; **c** Percent of liquid released and efficacy over varying surface areas for PBS-wetted control towelette; **d** Percent of liquid released and efficacy over varying surface areas for a 1.312% sodium hypochlorite product; **e** Percent of liquid released and efficacy over varying surface areas for a 1.4% hydrogen peroxide product; **f** Percent of liquid released and efficacy over varying surface areas for a 0.5% hydrogen peroxide product; **g** Percent of liquid released and efficacy over varying surface areas for a 0.21% quat product; **h** Percent of liquid released and efficacy over varying surface areas for a 0.28% quat product; **i** Percent of liquid released and efficacy over varying surface areas for a 0.76% quat + 22.5% alcohol product; **j** Percent of liquid released and efficacy over varying surface areas for a 0.308% quat + 21% alcohol product; **k** Percent of liquid remaining and efficacy over varying surface areas for 0.61% quat + 56% alcohol product

Data fitted Proc Glimmix with adequate robustness. Bacteria log_10_ reduction was significantly affected by disinfectant product (*p* < 0.0001), area (*p* = 0.0003), and strain (*p* = 0.0083). Two-level interactions disinfectant*area and disinfectant*strain were also significant (*p* = 0.0474 and *p* < 0.0001, respectively). Irrespective of area and strain, 0.55% sodium hypochlorite product (Fig. 1a) most effectively reduced bacterial load, while 0.5% quat + 55% alcohol product (Fig. 1b) was the least effective (both *p* < 0.0001). All of the disinfectants were significantly more bactericidal than PBS-wetted control wipe (*p* < 0.0001) (Fig. 1c). Notably, the sodium hypochlorite-based products (Fig. 1a and d), hydrogen peroxide-based (Fig. 1e and f), and quat-based products (Fig 1g and h) achieved a higher bactericidal efficacy than the quat alcohol-based products (*p* < 0.05) (Fig. 1i, j, k). Regardless of disinfectant product and strain, a higher log_10_ reduction value was reached when wiping one ft^2^ and two ft^2^ areas (*p* = 0.0006, *p* = 0.0015, respectively) compared to eight ft^2^. Aside from the PBS-wetted control wipe, 0.5% quat + 55% alcohol product (Fig. 1b) was the only disinfectant significantly impacted by area wiped. Specifically, the bactericidal efficacy for this product decreased as the area wiped increased. The 0.5% quat + 55% alcohol product was significantly more bactericidal when wiping one ft^2^ (*p* < 0.0001), two ft^2^ (*p* = 0.0166), and four ft^2^ (*p* = 0.0017) areas compared to eight ft^2^.

### Bactericidal efficacy varied between *P. aeruginosa* and *S. aureus* by disinfectant

Overall, all products were more effective against *P. aeruginosa* than *S. aureus* (*p* = 0.0083). While all disinfectants were more effective than the PBS-wetted control wipe in reducing both *P. aeruginosa* and *S. aureus* (*p* < 0.0001) (Fig. 1c), bactericidal efficacy differed among disinfectants for both strains tested. For *P. aeruginosa*, both sodium hypochlorite products achieved significantly higher log_10_ reduction values than most quat alcohol products. Specifically, the 1.312% sodium hypochlorite (Fig. 1d) and 0.55% sodium hypochlorite products (Fig. 1a) were significantly more effective than the 0.28% quat (Fig. 1h), 0.308% quat + 21% alcohol (Fig. 1j), 0.61% quat + 56% alcohol (Fig. 1k), and 0.5% quat + 55% alcohol products (Fig. 1b) (all *p* < 0.05). For *S. aureus*, the 0.5% quat + 55% alcohol product (Fig. 1b) was the least effective compared to all other disinfectants tested (all *p* < 0.05). Sodium hypochlorite, hydrogen peroxide and quat products yielded significantly higher log reduction than 0.308% quat + 21% alcohol and 0.5% quat + 55% alcohol products (*p* < 0.05).

### Percent of liquid released per ft^2^ from towelettes decreased as area wiped increased and varied among disinfectant products

Data adequately fit in the generalized linear mixed model. Overall, average liquid released per ft^2^ was significantly impacted by area wiped (*p* < 0.0001), disinfectant product (*p* < 0.0001), and their interaction (*p* < 0.0001). Greater amounts of liquid were released per ft^2^ on smaller areas wiped, compared to larger areas (Table 2). Specifically, greater liquid was released per ft^2^ when wiping the one ft^2^ area, compared to wiping all other areas respectively (*p* < 0.0001). More liquid was released per ft^2^ when wiping the two ft^2^ area, compared to wiping four, five, six, seven, and eight ft^2^ areas (all *p* < 0.0001). When wiping the three ft^2^ area, more liquid was released per ft^2^ compared to four, five, six, seven, and eight ft^2^ areas (all *p* < 0.0001). Wiping the four ft^2^ area released more liquid per ft^2^ than wiping the six, seven, and eight ft^2^ areas (all *p* = 0.001). Finally, wiping the five ft^2^ area released more liquid per ft^2^ compared to wiping seven ft^2^ (*p* = 0.0012) and eight ft^2^ areas (*p* < 0.0001).

**Table 2:** Average liquid released per ft^2^

Average liquid released per ft^2^ Average liquid released (g/ft^2^)^a^								
Towelette Product	1	2	3	4	5	6	7	8
0.55% sodium hypochlorite	0.51 ± 0.09	0.43 ± 0.08	0.40 ± 0.14	0.40 ± 0.07	0.34 ± 0.09	0.32 ± 0.08	0.37 ± 0.06	0.36 ± 0.05
0.5% quat + 55% alcohol	0.72 ± 0.54	0.38 ± 0.52	0.35 ± 0.04	0.29 ± 0.09	0.27 ± 0.02	0.24 ± 0.02	0.25 ± 0.06	0.22 ± 0.04
PBS-wetted towelettes	0.27 ± 0.06	0.22 ± 0.05	0.22 ± 0.08	0.20 ± 0.04	0.18 ± 0.03	0.17 ± 0.03	0.16 ± 0.03	0.16 ± 0.03
1.312% sodium hypochlorite	0.16 ± 0.16	0.33 ± 0.06	0.33 ± 0.03	0.27 ± 0.06	0.28 ± 0.03	0.25 ± 0.02	0.23 ± 0.01	0.21 ± 0.04
1.4% hydrogen peroxide	0.40 ± 0.21	0.26 ± 0.06	0.30 ± 0.01	0.21 ± 0.04	0.20 ± 0.03	0.20 ± 0.02	0.19 ± 0.03	0.18 ± 0.03
0.5% hydrogen peroxide	0.36 ± 0.12	0.26 ± 0.07	0.26 ± 0.05	0.22 ± 0.04	0.21 ± 0.04	0.19 ± 0.04	0.18 ± 0.03	0.17 ± 0.02
0.21% quat	0.32 ± 0.08	0.22 ± 0.05	0.22 ± 0.05	0.21 ± 0.06	0.21 ± 0.04	0.18 ± 0.03	0.17 ± 0.04	0.16 ± 0.05
0.28% quat	0.35 ± 0.12	0.24 ± 0.01	0.25 ± 0.06	0.19 ± 0.06	0.17 ± 0.03	0.16 ± 0.01	0.16 ± 0.02	0.16 ± 0.03
0.76% quat + 22.5% alcohol	0.50 ± 0.12	0.31 ± 0.04	0.27 ± 0.04	0.23 ± 0.03	0.22 ± 0.02	0.21 ± 0.03	0.20 ± 0.02	0.19 ± 0.02
0.308% quat + 21% alcohol	0.25 ± 0.03	0.19 ± 0.02	0.17 ± 0.01	0.16 ± 0.01	0.15 ± 0.01	0.14 ± 0.01	0.13 ± 0.01	0.14 ± 0.01
0.61% quat + 56% alcohol	0.72 ± 0.39	0.32 ± 0.09	0.27 ± 0.09	0.19 ± 0.04	0.18 ± 0.04	0.16 ± 0.01	0.15 ± 0.01	0.15 ± 0.05

^a^Average liquid released per ft^2^ was calculated by measuring total liquid released (measured in g) onto a total area wiped divided by the number of ft^2^ wiped

There was a greater amount of liquid released per ft^2^ from the sodium hypochlorite-based products than quat-, quat-alcohol-, and hydrogen peroxide-based products (*p* < 0.05). Notably, the 1.312% sodium hypochlorite-based product released less liquid per ft^2^ compared to the 0.55% sodium hypochlorite product (*p* < 0.0001). Hydrogen peroxide-based products released more liquid per ft^2^ than 0.28% quat product, but less compared to 0.5% quat + 55% alcohol product (*p* < 0.05). The 0.308% quat + 21% alcohol product released less liquid per ft^2^ compared to the other quat alcohol products, quat-based products, and hydrogen peroxide-based products (all *p* < 0.0001). Additionally, 0.76% quat + 22.5% alcohol and 0.5% quat + 55% alcohol products released more liquid per ft^2^ than 0.21% quat product (*p* < 0.0001). The 0.76% quat + 22.5% alcohol, 0.61% quat + 56% alcohol, and 0.5% quat + 55% alcohol products released more liquid per ft^2^ than the 0.28% quat product (*p* < 0.05). The 0.61% quat + 56% alcohol product released less liquid per ft^2^ compared to the 0.5% quat + 55% alcohol product (*p* < 0.0001).

The interaction affect between disinfectant and area was significantly (*p* < 0.0001). The amount of liquid released per ft^2^ decreased as the area wiped increased among all of the disinfectant products tested (*p* < 0.05). Across all disinfectant products, there was a significantly greater liquid released per ft^2^ when wiping one ft^2^ compared to eight ft^2^ (*p* < 0.05).

## Discussion

In this study we tested ten RTU disinfectant towelette products to determine the impact of surface area wiped on bactericidal efficacy using quantitative methodology. Bactericidal efficacy varied among RTU towelette products tested, the size of surface wiped, and by strain. Further, we found significant differences in the percentage of liquid released per ft^2^ among wipes across various surface areas. There is not, to our knowledge, any peer-reviewed literature currently published that has examined the impact of surface area on a disinfectant’s bactericidal efficacy. We believe this is the first study to use the EPA disinfectant towelette methodology in scenarios relevant to healthcare facilities.

### Bactericidal efficacy varies by towelette product and total surface area wiped

There were statistically significant differences in bactericidal efficacy among the ten disinfectant towelette products tested. Overall, the 0.5% quat+ 55% alcohol product had the lowest bactericidal efficacy compared to all other tested products. The 0.55% sodium hypochlorite product tested achieved the highest bactericidal efficacy. This is consistent with our prior work [5] and other published literature [17, 18]. We elected to test bactericidal efficacy at one, two, four, and eight ft^2^ as opposed to all continuous ft^2^ based on preliminary data that indicated differences in efficacy was marginal among some surface areas. This was also consistent with the percent liquid released per ft^2^ where there was no significant differences in percent released per ft^2^ among the six, seven, and eight ft^2^ areas.

The 0.5% quat + 55% alcohol product achieved the lowest bactericidal efficacy overall and across all surface areas tested for *S. aureus*, with only an ~ 3 log_10_ reduction achieved after wiping the eight ft^2^ area. However, this product released one of the highest percentages of liquid per ft^2^. This inverse relationship may indicate that although liquid is being released from the disinfectant towelette, the liquid may not contain enough active ingredients to achieve the ~ 5–6 log_10_ reduction performance standard [19] for *S. aureus*. Although previous studies have shown that the addition of other active ingredients with bactericidal properties to quat-based products enhances their bactericidal efficacy [20]), our results indicate the opposite. It is worth noting that quat-based disinfectant products are reported to be among the most commonly used in healthcare facilities for cleaning surfaces [21, 22], although some products such as the 0.5% quat + 55% alcohol product tested in this study may not be achieving a ~ 5–6 log_10_ reduction for all bacterial species. These results might also be due to the high alcohol content of this product. The alcohol may be evaporating too quickly to achieve the ~ 5–6 log_10_ kill needed to meet the performance standard guidelines [19, 23–25].

### Towelettes were less effective as surface area increased, which may have implications for disinfection of large surfaces

Overall, there was a higher log reduction achieved when wiping the one and two ft^2^ surface areas compared to the eight ft^2^ surface area. Although the extent to which bactericidal efficacy is impacted is product dependent, it indicates that wiping a larger surface will lead to reduced bactericidal efficacy. The current EPA testing requirements for product registration do not consider varying surface areas. In fact, only a small glass slide is tested in the current protocol, which is not representative of a large surface area (such as a countertop) that would be wiped in a healthcare setting. Additionally, towelette bactericidal efficacy is interpreted based on the EPA’s product performance test guidelines (OCSPP 810.2200) for disinfectants used on environmental surfaces [19]. For the AOAC Germicidal Spray Products as Disinfectants test and towelette methods (used for hospital disinfectant validation), “the product should kill all of the test microorganisms on 59 out of each set of 60 carriers”, which is repeated three times with starting carrier inoculums of ~ 5–6 log bacteria) [14]. Multiple products tested narrowly made or did not meet a 5–6 log reduction performance standard when tested on larger surface areas. This indicates a need for the performance standards to include larger surface areas in disinfectant towelette validation testing, which is more “real-world applicable” to how the towelettes are used in the healthcare industry.

We noted during our wiping process that certain towelettes became harder to move across the Formica surface as the surface area became larger. This was particularly noticeable when wiping with the 0.55% sodium hypochlorite towelette, which had a lower percentage of liquid released per ft^2^ overall yet achieved the highest bactericidal efficacy. Taken together, we hypothesize that the towelette itself, as it becomes dryer, is physically removing bacteria from the surface via friction and contact. Conversely, we suggest the 0.5% quat + 55% alcohol towelette, which released a higher percentage of liquid per ft^2^, may have allowed it to “glide” over the surface thus reducing physical removal of microorganisms. The PBS-wetted control towelette achieved an approximate two log reduction overall. The PBS control results are similar to Rutala et al., who investigated the efficacy of a non-germicidal product against *Clostridium difficile* spores on Formica surfaces [26]. They found that physical removal via wiping led to a three log reduction in spores from environmental surfaces [26]. This further substantiates that the towelette substrate and physical wiping motion are contributing to microbial reduction.

### Disinfectant efficacy is bacterial species-dependent

Overall, disinfectant towelettes were more effective against *P. aeruginosa* than *S. aureus. P. aeruginosa* was reduced a range of 0.12–0.80 log_10_ more than *S. aureus*. The CDC’s Guidelines for Disinfection and Sterilization in Healthcare Facilities acknowledges that disinfectant efficacy can vary depending on the target microorganism [27]. Our prior work on bactericidal efficacy of products against *P. aeruginosa* versus *S. aureus* [5] and study by Hong [7] are consistent with the findings in this study. This implies the need to investigate more strains and species in future research to determine the full implications of species-dependent differences. We acknowledge that our study was limited to two bacterial species and only one strain of each species underscoring the need for further work. Testing was also done using pure bacterial cultures of each species, per EPA methodology [15]. Therefore, we cannot determine the impact a mixed bacterial culture or more complex matrices such as a biofilm would have on disinfectant products’ efficacies.

## Conclusions

Overall, a disinfectant towelette bactericidal efficacy varies by product used, size of surface area wiped, and by target bacterial species. The results in this study indicate a clear need for further research to determine the efficacy constraints of these products, particularly as they become more frequently used in health care settings.

## Abbreviations

EPA: Environmental Protection Agency
HAI: Healthcare-associated infection
PBS: Phosphate buffered saline
Quat: Quaternary ammonium compounds
RTU: Ready-to-use
